# Induction of Tolerogenic Dendritic Cells by a PEGylated TLR7 Ligand for Treatment of Type 1 Diabetes

**DOI:** 10.1371/journal.pone.0129867

**Published:** 2015-06-15

**Authors:** Tomoko Hayashi, Shiyin Yao, Brian Crain, Victor J. Promessi, Luke Shyu, Caroline Sheng, McNancy Kang, Howard B. Cottam, Dennis A. Carson, Maripat Corr

**Affiliations:** 1 Moores Cancer Center, University of California San Diego, 9500 Gilman Drive, La Jolla, CA, 92093-0695, United States of America; 2 Department of Medicine, University of California San Diego, 9500 Gilman Drive, La Jolla, CA, 92093-0663, United States of America; University of Leuven, Rega Institute, BELGIUM

## Abstract

Autoimmune diabetes mellitus (DM) results from the destruction of pancreatic islet cells by activated T lymphocytes, which have been primed by activated dendritic cells (DC). Individualized therapy with *ex vivo* DC manipulation and reinfusion has been proposed as a treatment for DM, but this treatment is limited by cost, and requires specialized facilities. A means of *in situ* modulation of the DC phenotype in the host would be more accessible. Here we report a novel innate immune modulator, 1Z1, generated by conjugating a TLR7 ligand to six units of polyethylene glycol (PEG), which skews DC phenotype *in vivo*. 1Z1 was less potent in inducing cytokine production by DC than the parent ligand *in vitro* and *in vivo*. In addition, this drug only modestly increased DC surface expression of activation markers such as MHC class II, CD80, and CD86; however, the expression of negative regulatory molecules, such as programmed death ligand 1 (PD-L1), and interleukin-1 receptor-associated kinase M (IRAK-M) were markedly increased. *In vivo* transfer of 1Z1 treated DC into prediabetic NOD mice delayed pancreatic insulitis. Daily administration of 1Z1 effectively prevented the clinical onset of hyperglycemia and reduced histologic islet inflammation. Daily treatment with 1Z1 increased PD-L1 expression in the CD11c^+^ population in peri-pancreatic lymph nodes; however, it did not induce an increase in regulatory T cells. Pharmaceutical modulation of DC maturation and function *in situ*, thus represents an opportunity to treat autoimmune disease.

## Introduction

Dendritic cells (DC) contain heterogeneous populations that contribute to initiating and perpetuating inflammatory processes as well as immunologic tolerance [1]. Various Toll-like receptor (TLR) stimuli contribute to maturation of DC leading to either initiation of immune responses or to tolerance [2, 3]. Indeed, tolerogenic DC may be needed to maintain the immune homeostasis in peripheral tissues [4]. Initially, the ability of DC to induce tolerance was demonstrated with immature DC located in the peripheral lymphoid organs [2]. More recent studies indicated that a tolerogenic DC population produces negative regulatory factors, such as IL-10 and indoleamine 2, 3-dioxygenase (IDO), without inducing proinflammatory cytokines under steady state conditions [4–6]. In addition, increased surface expression of negative regulatory co-stimulatory molecules, like program death-ligand 1 (PD-L1), on tolerogenic DC contributes to reducing effector T cell differentiation and activation in favor of inducing regulatory T cells (Treg) [7, 8].

One mechanism of stimulating DC maturation is through Toll-like receptor (TLR) stimulation. These innate immune receptors sense and identify danger signals, and initiate immune defense reactions in the hosts. However, TLRs also respond to endogenous ligands such as those released by dying tissues. The release of these endogenous TLR agonists has been suggested as a mechanism of perpetuating inflammation in autoimmune diseases and TLR7 has been particularly implicated, making this TLR a candidate for targeted therapy [9]. In addition TLR7 responds to synthetic low molecular weight ligands, including imidazoquinolines, and purine-like molecules [10–12]. We previously demonstrated that daily administration of a specific TLR7 ligand, 9-benzyl-8-hydroxy-2-(2-methoxyethoxy) adenine (1V136) [13], could reduce autoimmune disease and modulate DC function [14, 15]. To potentially lengthen the *in vivo* efficacy of 1V136, our laboratory modified the parent compound by click chemistry to link it to a six unit oligo-ethylene glycol (PEG) moiety and a carboxyl tail [16]. The new compound, 1Z1, retained TLR7 specificity, had a lower stimulatory potency than the parent compound, and also attenuated TLR7 activation and non-specific inflammation [17]. Hence, preliminary investigations suggested that 1Z1 had desirable functional properties as an anti-inflammatory agent that could be used to abate the onset of autoimmune disease or potentially limit the progression and end organ destruction.

In this report, we tested the ability of the PEGylated compound to ameliorate the course of spontaneous diabetes in non-obese diabetic (NOD) mice. Here we demonstrate that DC treated *ex vivo* with the PEGylated derivative, 1Z1, and injected into NOD mice delayed the onset of insulitis, suggesting that 1Z1 treated DC were functionally tolerogenic. More importantly, repeated treatment with 1Z1 *in vivo* prevented diabetic onset in NOD mice. The clinical efficacy was associated with an increase in PD-L1 expression on DC in the draining pancreatic lymph nodes, but not in distant lymphoid organs. These data indicate that 1Z1 could be a new class of treatment for diabetes and possibly other autoimmune diseases, by safely and selectively inducing DC to express PD-L1 in areas of inflammation.

## Materials and Methods

### Mice

7–8 week old female NOD mice and C57BL/6 mice were purchased from The Jackson Laboratory (Bar Harbor, MA). Female mice were used in this study as they more reliably develop diabetes in standard housing conditions. *Tlr7* ^*-/-*^ mice were a gift from Dr. S. Akira (Osaka University, Osaka, Japan) and bred onto the C57BL/6 background at University of California, San Diego (UCSD).

### Ethics Statement

This study was carried out in strict accordance with the recommendations in the Guide for the Care and Use of Laboratory Animals of the National Institutes of Health. The protocol was approved by the Institutional Animal Care and Use Committee of University of California, San Diego (PHS Animal Welfare Assurance Number: A3033-01; Protocol Numbers: S00028 and S00060). Mice were sacrificed by CO_2_ inhalation followed by cervical dislocation. All efforts were made to minimize suffering during the procedures in this project.

### Reagents

1Z1, the PEGylated TLR7 ligand and the reference TLR7 agonist (1V136), were synthesized in our laboratory [16, 18] and dissolved in DMSO as 100 mM stock solutions and kept at -20°C until use. Endotoxin levels of these drugs were <10 EU/μmol as determined by Endosafe. (Charles River laboratory, Wilmington, MA). The stock solutions were diluted in normal saline with a final DMSO concentration of 0.5%, which was also used as the vehicle control. RPMI 1640 medium, and DMEM (both from Life Technologies, Carlsbad, CA) were supplemented with 10% FCS and penicillin/streptomycin (both from Sigma Chemical Co., St Louis, LA) to make complete RPMI or complete DMEM.

#### In vitro generation and experiments of bone marrow derived cells and splenocytes

Bone marrow derived dendritic cells (BMDC) or macrophages (BMDM) were prepared from C57BL/6 or NOD mice as previously described [19, 20]. BMDC (1×10^5^ cells per well) and BMDM (5 ×10^4^ cells per well) were plated in 96-well plates in triplicate in 200 μl complete RPMI 1640 and complete DMEM, respectively. The cells were incubated with graded concentrations of the compounds for 18 h at 37°C, 5% CO_2_. After 18 h incubation, the cell culture supernatants were collected. The levels of IL-6, IL-10 or IL-12 in the culture supernatants were determined by ELISA (BD Biosciences, La Jolla, CA). For B cell proliferation assays, splenocytes isolated from C57BL/6 were incubated with 10 μM carboxyfluorescein succinimidyl ester (CFSE) and washed. CFSE labeled cells were cultured with 1Z1 or 1V136 for 5 days and stained for B220^+^ B cells. Cell proliferation was monitored by CFSE dilution using FACSCanto flow cytometer (BD Bioscience) and analyzed using FlowJo software (Tree Star, Ashland, OR). Cell division was quantified by the proliferative index (PI = the sum of the cells in all generations / the number of original parent cells) and the % proliferation (number of calculated parent cells that underwent proliferation)[21]. BMDC were cultured with 1Z1 or vehicle overnight and stained with antibodies against CD40, CD80, CD86, or MHC class II (eBiosciences, San Diego, CA). Expression of CD40, CD80, CD86, or MHC class II in the gated CD11c^+^ population was studied using FACSCanto flow cytometer and FlowJo software.

### T cell isolation and co-culture with BMDC 1Z1 treatment in vitro

C57BL/6 mice were immunized with ovalbumin (Worthington Biochemical Corporation, Lakewood, NJ) mixed with ODN1860 (50 μg/mouse) day 0 and 14. CD4^+^ T cells were isolated from splenocytes harvested from mice immunized with OVA plus ISS-ODN on days 0 and 14, using EasySep Mouse CD4 Positive Selection Kit (STEMCELL Technologies, Vancouver, BC. Canada). CD4^+^ T cells (2×10^5^/well of a 96-well plate) were labeled with carboxyfluorescein succinimidyl ester (1 μM, CFSE) for 30 min at 37°C, washed and then cultured with OVA (10 μg/mL) and BMDC (2 × 10^5^/well of a 96-well plate) in the presence of 1Z1 (1 μM, or 5 μM) or vehicle in triplicate for 3 days. Cell proliferation was monitored by CFSE dilution with flow cytometry.

### Activity assay of 1Z1 by human TLR7 reporter cells and human peripheral blood mononuclear cells

Human TLR7, NF-κB/SEAPorter HEK 293 cells (Imgenex catalog# IML-107, San Diego, CA) (5 × 10^4^ cells per well of 96 well plate) were incubated with graded doses of 1Z1. The culture supernatants were harvested after a 20–24 h incubation period. Secreted alkaline phosphatase (SEAP) activity in the supernatants was determined by a colorimetric assay, using the SEAPorter Assay Kit (Imgenex, San Diego, CA), with absorbance read at 630 nm. Human peripheral blood mononuclear cells (PBMC) were isolated from the buffy coats purchased from San Diego Blood Bank using Ficoll-paque gradient centrifugation [18]. PBMC (10^6^ /mL) were co-cultured with 1Z1 or 1V136 for 18 h and IFNα1, IL-6, IL-12, TNFα, or IL-1β in the culture supernatants were measured by Luminex beads assay (Millipore, Billerica, MA).

### Response of CD4^+^ T cells to TLR7 ligands and in vitro functional assay of regulatory T cells

CD4^+^ T cells were isolated from splenocytes of C57BL/6 or NOD mice using a mouse CD4 positive selection kit (Stemcell Technologies Inc, Vancouver BC, Canada). CFSE-labeled CD4^+^ T cells (10^5^/ well of 96 well plate) were cultured with 1Z1 (1, 2, 5, or 10 μM), LPS (10 ng/mL), or IL-2 (10 unit/mL) in the presence and absence of plate coated anti-CD3 monoclonal antibody (αCD3) for 3 days. After 3 days culture, the cells were stained for CD4 and CD25. Cell proliferation was monitored by CFSE dilution in CD4^+^, CD4^+^/CD25^+^, or CD4^+^/CD25^-^ gated population. To test whether the repeated 1Z1 treatment influences the suppressor function of regulatory T cells (Treg) *in vivo*, NOD mice were treated with 1Z1 (400 nmol/animal) for 7 days and splenocytes were harvested. CD4^+^CD25^+^ Treg and CD4^+^CD25^-^ conventional T cells (Tconv) were isolated using EasySep Mouse CD4^+^CD25^+^ Regulatory T Cell Isolation Kit (Stemcell Technologies Inc.). CFSE-labeled Tconv (10^5^ cells per 200-μl in a 96 well plate) were cultured with anti-CD3 and anti-CD28 antibody coated beads (2.5 × 10^5^/mL, Dynabeads Mouse T-Activator CD3/CD28 for T-Cell Expansion and Activation, Life Technologies). Unlabelled Treg cells (2 × 10^4^) were added to selected wells, and co-cultured for 3 days. Cell proliferation was monitored by CFSE dilution in the CD4^+^ gated population and the proliferation index and %proliferation were calculated as above.

### Systemic cytokine release following subcutaneous injection of 1V136 or 1Z1

C57BL/6 and NOD mice (n = 3–4/group) were subcutaneously (s.c.) injected with 200, 400 or 600 nmol 1V136 or 1Z1 in 100 μL vehicle (6% DMSO in saline). Two hours after the injection, sera were collected and the levels of TNFα, IL-6, and IL-12 in the sera were determined by Luminex beads assay (Life Technologies).

### 1Z1 treatment and diabetes monitoring

Female NOD mice (n = 5 to 10 /group) were daily treated with vehicle, or 1Z1 400 nmol [17] from week 8–9 for 4 weeks or 18 weeks. Glycosuria was evaluated weekly using Diastix (Bayer, Elkhart, IN). When glycosuria (glucose levels in urine>250 mg/dL) was detected, blood glucose levels were determined by OnetouchUltra (Lifescan, Milpitas, CA). Hyperglycemia (>300 mg/dL) was confirmed by additional evaluations of blood glucose levels 24 h later. Diabetes was diagnosed by two consecutive positive tests for hyperglycemia. In some experiments, body weight was monitored daily. In another experiment, mice were sacrificed after a 4-week treatment and pancreatic inflammation was histologically scored as previously described [22]. Briefly, pancreases were harvested and stained with hematoxylin and eosin (H&E) by the UCSD Histology Core. Ten to 40 islets from each pancreas were scored. The severity was determined according to the following criteria; no infiltration (grade 0), perivascular/periductular infiltrates with leukocytes touching islet perimeters, but not penetrating (grade 1), leukocytic penetration of up to 25% of islet mass (grade 2), leukocytic penetration of up to 75% of islet mass (grade 3), end-stage insulitis, <20% of islet mass remaining (grade 4). Insulitis index was calculated as I = [(0×n_0_) + (1×n_1_)+ (2×n_2_)+ (3×n_3_)+ (4×n_4_)] / 4 × (n_0_ + n_1_+ n_2_ + n_3_ + n_4_), where n_0_, n_1_, n_2_, n_3_, and n_4_ were the number of islets scored in grade 0, 1, 2, 3, 4, respectively [22]. Sera were collected 2h after the initial and 7^th^ injections, and levels of IL-6 and TNFα were determined by Luminex bead assay. Single cell suspension of pancreatic lymph nodes and spleens were prepared using collagenase/DNase digestion. Single cell suspension of pooled pancreatic lymph node cells, splenocytes or buffy coats from treated NOD mice were stained for CD3^+^, CD4^+^ and CD8^+^, CD11c^+^, PD-L1^+^, CD69^+^ or CD25^+^ and intracellular Foxp3.

### Adoptive transfer experiments of 1Z1 treated DC to NOD mice

BMDC were prepared from 8–9 week old female NOD mice. BMDC were incubated with 1Z1 or vehicle overnight and washed three times with medium. 1Z1- or vehicle- treated cells were intravenously transferred into 8 week old female NOD mice. Four weeks after the adoptive transfer, mice were sacrificed and histologic examination of the pancreatic tissues was performed as described above. In some experiments, NOD BMDC were incubated with the islet peptide GAD65_515–524_ (100 μg/mL) [23, 24] with or without 1Z1 for 5 hours and then transferred into 8 week old female NOD mice (4/group). Vehicle treated DC and 1Z1 treated DC were used as controls. Insulitis onset was evaluated 9 weeks after cell transfer after at least one mouse in the naïve group developed diabetes to ensure that the experiment would not be prematurely terminated.

### Quantitative RT-PCR

Total RNA was extracted from tissues or cells. Harvested tissues were immediately frozen in liquid nitrogen and stored at -80°C. Total RNA was isolated using RNeasy Mini Kit (Qiagen). cDNA synthesis was performed using the iScript (Bio-Rad) and real time PCR were performed on the Bio-Rad iCycler IQ. The comparative ^ΔΔ^Ct method was used to assess fold changes in expression of RNA transcripts between control and drug-treated mice. ^Δ^Ct values were determined by subtracting the average GAPDH RNA gene Ct values from each test Ct value. ^ΔΔ^Ct values were normalized by the control calibrator ^Δ^Ct value. Taqman Gene Expression Assays (murine IRAK-M, IL-10 and PD-L1) used for the qPCR analysis were purchased from Life Technologies.

### Immunoblotting

BMDM (10^6^) were stimulated with 10 μM 1Z1 or vehicle overnight. Cell lysates were separated on a 4 to 12% SDS-PAGE gel (Thermo Fisher Scientific). After the proteins were transferred onto a PVDF membrane, and probed with an anti-IRAK-M antibody (Ab8116, Abcam, Cambridge, MA) and developed by ECL (Thermo Fisher Scientific). The blot was then probed with an anti-actin antibody (C4, Millipore, Billerica, MA).

### Statistical analysis

Prism 6.0 (GraphPad, San Diego CA) was used for statistical analyses including regression analyses. Data were plotted and fitted by nonlinear regression assuming a Gaussian distribution with uniform standard deviations between groups and EC_50_ was calculated. The data are reported as means ± standard errors of the mean (SEM). The Mann—Whitney U test was used to compare two groups. One-way ANOVA with Bonferroni or Dunnett’s *post hoc* test was used for multiple comparisons to a control group. Kaplan-Meier Survival Analysis with a log-rank test was used to monitor the incidence of diabetes.

## Results

### 1Z1 reduces pro-inflammatory properties *in vitro and in vivo*

**Fig 1:**
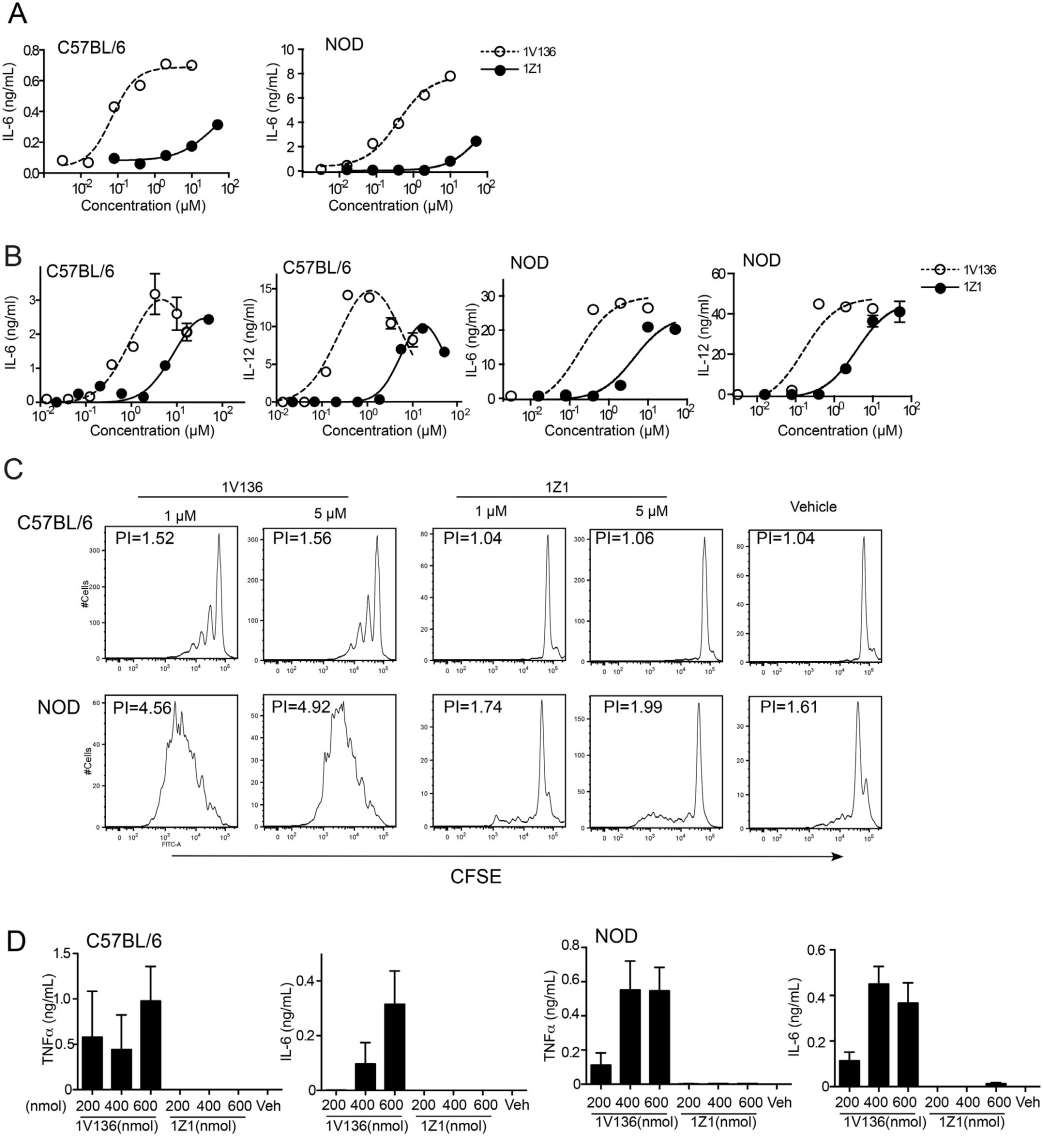
PEGylated TLR7 ligand 1Z1 lacks proinflammatory activities *in vitro* and *in vivo*. (A) Murine splenocytes from C57BL/6 and NOD mice (1 × 10^5^/well of a 96 well plate) were incubated with graded doses of 1Z1 or 1V136 in triplicate of each concentration for 18 h and IL-6 was measured by ELISA. Data are average ± SEM of triplicates and are representative of 3 independent experiments. (B) BMDC from C57BL/6 and NOD mice were plated (1× 10^5^/well) were incubated with graded doses of 1Z1 or 1V136 in triplicate of each concentration for 18 h and IL-6 and IL-12 were measured by ELISA in the culture supernatants. Data are average ± SEM of triplicates and are representative of 3 independent experiments. (C) Splenocytes isolated from C57BL/6 and NOD mice were incubated with indicated concentrations of 1Z1 or 1V136 for 3 days. B cell proliferation was evaluated by CFSE dilution in the gated B220^+^ population. Proliferative indexes (PI) were calculated and are shown in the panel, which is representative of 3 independent experiments. (D) C57BL/6 or NOD mice were s.c. injected with 200, 400 or 600 nmol 1V136 or 1Z1 (n = 3–4 mice per group). Two hours after the injection, the levels of TNFα, and IL-6 in the sera were determined. The averages of the cytokine levels in the sera are shown ± SEM.

The bioactivity of the PEGylated compound 1Z1, was compared to the parent compound, 1V136 *in vitro*. Splenocytes from C57BL/6 and NOD mice containing mixed cell populations were stimulated with graded doses of each compound and IL-6 production was measured. 1Z1 was 1000 to 100 times less potent than 1V136 for IL-6 stimulation (C57BL/6: EC_50 (1Z1)_ = 75 μM vs. EC_50(1V136)_ = 0.07 μM, NOD: EC_50(1Z1)_ = 73 μM vs. EC_50(1V136)_ = 0.4 μM) (Fig 1A). Using bone marrow-derived dendritic cells (BMDC), the IL-6 or IL-12 production induced by 1Z1 was about 10 fold attenuated compared to 1V136 for both C57Bl/6 and NOD derived cells (Fig 1B). Since B cells also express TLR7 and are a major source of IL-6 production in a splenocyte population, we evaluated the effect of 1Z1 on B cell proliferation using CFSE labeling (Fig 1C). The parent compound 1V136 induced B220^+^ B cell proliferation at doses as low as 1 μM in B cells from C57BL/6 and NOD mice. In addition, B cells from NOD mice (PI = 4.52 at 1 μM) underwent three times more cell divisions compared to C57BL/6 (PI = 1.52 at 1 μM). In contrast, minimal proliferation was induced by 1Z1 at 5 μM in both strains (Fig 1C).

To further examine the immune properties of 1Z1 *in vivo*, 1Z1 was subcutaneously injected to C57BL/6 or NOD mice and cytokine levels in sera were measured 2 h post administration (Fig 1D). In C57BL/6 mice, The highest dose of 1Z1 (600 nmol per animal) did not induce TNFα or IL-6 *in vivo*, whereas 1V136 administration resulted in a substantial cytokine release at all doses. In the dose response there was a small, but detectable level of IL-6 in the sera of NOD mice that were injected with 600 nmol per animal 1Z1. Hence, 400 nmol per dose of 1Z1 was selected for further study as the highest dose in NOD mice that did not result in measurable systemic cytokine release.

**Fig 2:**
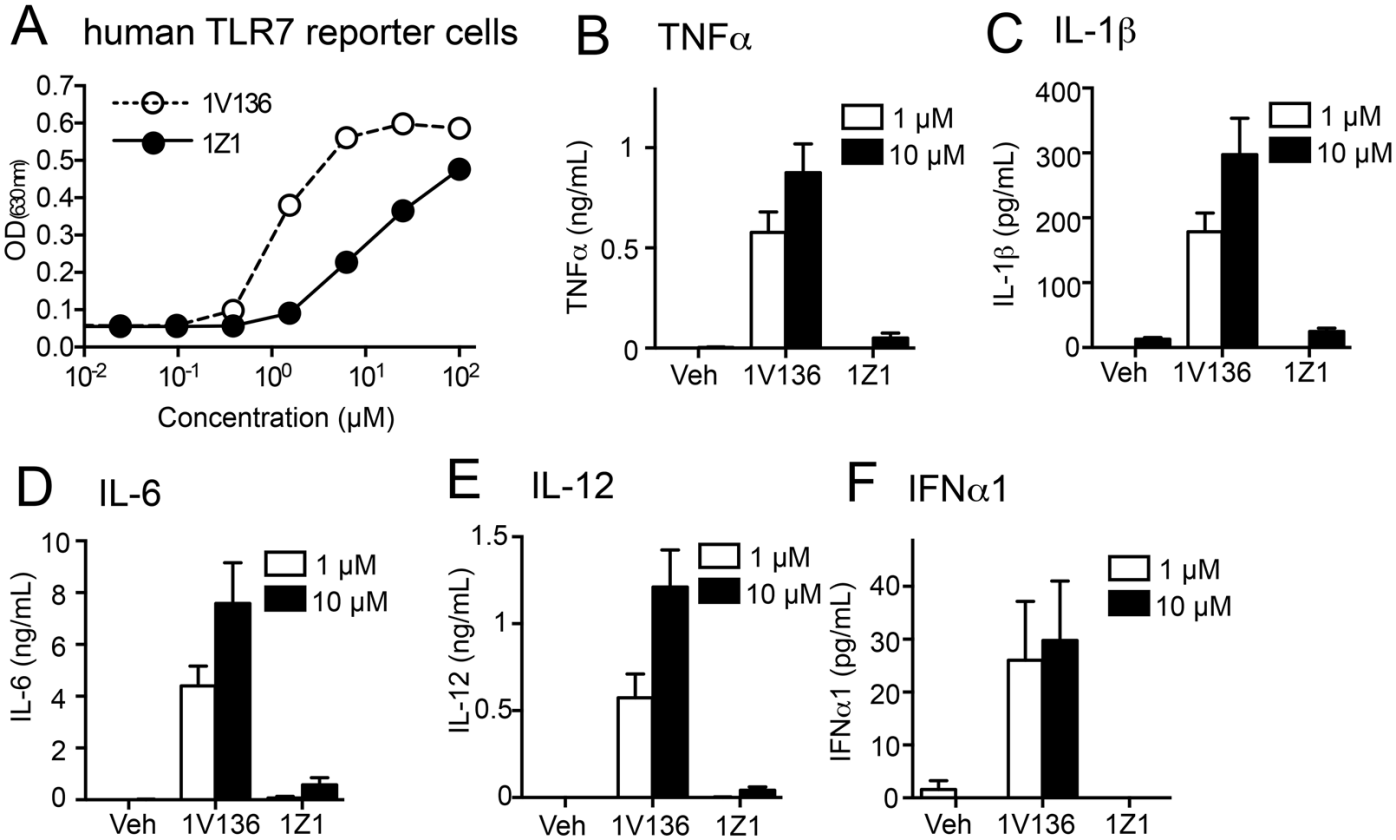
1Z1 has diminished ability to stimulate cytokine secretion in human PBMC. (A) Human TLR7-NF-κB HEK293 reporter cells were incubated with graded concentrations of 1V136 or 1Z1 in triplicate for each concentration and the levels of NF-κB translocation were measured by SEAP activity in the culture medium (OD_630_). Data shown are averages ± SEM of triplicates and are representative of two independent experiments. (B-F) Human PBMC were cultured with 1Z1 or 1V136 for 18 h. The levels of TNFα (B), IL-1β (C), IL-6 (D), IL-12 (E), and IFNα1 (F) in the culture supernatants were determined by Luminex bead assay. Data shown are mean ± SEM of 3 independent donors.

Future therapeutic development of 1Z1 and other attenuated TLR7 ligands would require that these compounds retain their ability to interact with human TLR7. Human embryonic kidney 293 (HEK293) cells with a stable NF-κB reporter that expresses secreted embryonic alkaline phosphatase (SEAP) when stimulated through human TLR7, was therefore treated with 1V136 and 1Z1. The 1Z1 treated reporter cells produced detectable but low SEAP release compared to 1V136 treated cells, indicating that this compound retained the ability to stimulate human TLR7, albeit at a lower potency (Fig 2A). The effects of 1Z1 were also tested on human peripheral mononuclear cells (hPBMC). Induction of proinflammatory cytokines (TNFα, IL-1β, IL-6, and IL-12) and type I IFN by 1 and 10 μM 1Z1 was significantly lower than the concentrations induced by 1V136 (Fig 2B–2F).

### 1Z1-treated DC display a semi-mature phenotype and functionally suppress T cell activation *in vitro and in vivo*

**Fig 3:**
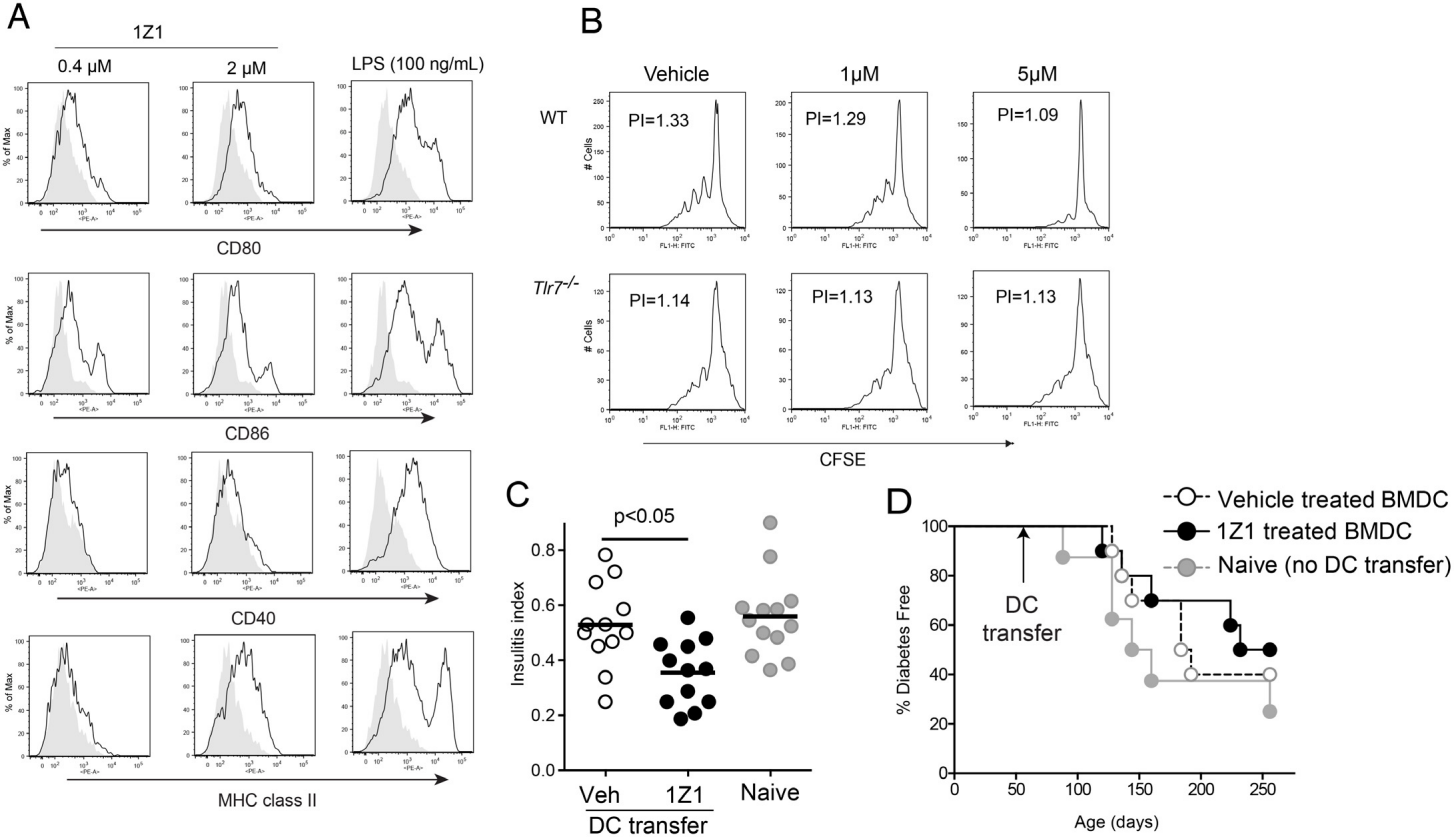
1Z1 treated DC are semi-matured, inhibit proliferative T cell responses and delay the onset of diabetes. (A) BMDC prepared from NOD mice (A) were incubated with vehicle, 1Z1 (0.4 and 2 μM) or LPS (100ng/mL) overnight. The expression levels of CD80, CD86, CD40 or MHC class II, in the gated CD11c^+^ population are shown (solid black line). The expression level on vehicle treated cells is shown in shaded gray. (B) OVA primed CD4^+^ T cells were labeled with CFSE and incubated with WT or *Tlr7* ^*-/-*^ BMDC, with OVA and 1Z1 (1 or 5 μM) or vehicle for 5 days. T cell proliferation was monitored by CFSE dilution. Data shown are representative of 2 independent experiments that had similar results. (C) BMDC prepared from NOD mice were treated with 2 μM 1Z1 or vehicle overnight. 2 × 10^6^ cells were intravenously transferred into 8 week old NOD mice. Four weeks after the BMDC transfer, the pancreases were histologically prepared and stained with H&E. The severity of insulitis was expressed as insulitis indexes. Data are pooled from two independent experiments. (D) Incidence of diabetes in NOD mice (n = 8–10 total /group) recipients of naïve BMDC, 1Z1- or vehicle-treated BMDC. The onset of glucosuria as a marker of diabetes was evaluated weekly by urine glucose levels. When glycosuria was detected, blood glucose levels were determined. The hyperglycemia (>300 mg/dL) was confirmed by additional evaluations of blood glucose levels 24 h later. The diabetes was diagnosed by two consecutive positive tests for hyperglycemia.

To evaluate the immunologic characteristics of 1Z1 treated BMDC, the surface expression of co-stimulatory molecules was evaluated by flow cytometric assay. Compared to the lipopolysaccharide (LPS) treated or reference TLR7 ligand, 1V136, treated cells, 1Z1 induced only minimal increases in expression of CD80, CD86, CD40 and MHC class II at 0.4 and 2 μM (Fig 3A). However, at a high dose (5 μM) the level of CD80 and CD86, but not CD40 were as high as that seen with cells stimulated with 1 μM 1V136 (S1 Fig).

We then tested the effect of 1Z1 treated DC on clonal expansion of T cells *in vitro*. WT C57BL/6 or TLR7 null BMDC were incubated with ovalbumin (OVA)-primed CD4^+^ T cells and OVA in the presence of 1Z1 or vehicle. The vehicle treated cultures showed a robust T cell proliferation as monitored by CFSE (Fig 3B). However, 1Z1 exposure reduced proliferation of antigen specific CD4^+^ T cells in a dose dependent manner (Fig 3B). 1Z1 was not effective in inhibiting T cell expansion in cultures with TLR7 null DC and WT T cells, indicating that the effect was dependent on TLR7 on the BMDC, and not on the T cells (Fig 3B).

Next, to evaluate the function of 1Z1 treated DC *in vivo*, we used a murine model of type 1 diabetes, the NOD mouse strain. In this murine model, DC based therapy is known to be effective for prevention or treatment of disease [23–30]. We chose 2μM 1Z1 as the dose that demonstrated only a minimal response in the *in vitro* studies (Fig 1A and1B) to generate a tolerogenic phenotype (Fig 3A). Two million BMDC that were pretreated with 2 μM 1Z1 or vehicle overnight *in vitro* were adoptively transferred into 8 week-old prediabetic NOD mice. Four weeks after the transfer, the severity of insulitis was evaluated histologically in pancreatic sections. The pancreases from NOD mice that received 1Z1-treated BMDC had s4nificantly lower disease severity indexes than mice that received vehicle-treated or naïve BMDC (Fig 3C). These findings indicated that 1Z1 pretreated BMDC were functionally suppressive *in vivo*. However, the suppression of insulitis by 1Z1 treated BMDC was not sustained and did not prevent diabetes after a longer observation period (Fig 3D). The addition of a known islet peptide, GAD_515–524_ [23, 24] to the 1Z1 treated DC resulted in accelerated histologic insulitis rather than greater tolerogenicity (S2 Fig). The results suggested suppression of diabetes would require continual intervention with 1Z1.

### 1Z1 induces an innate immune suppression signature in DC

**Fig 4:**
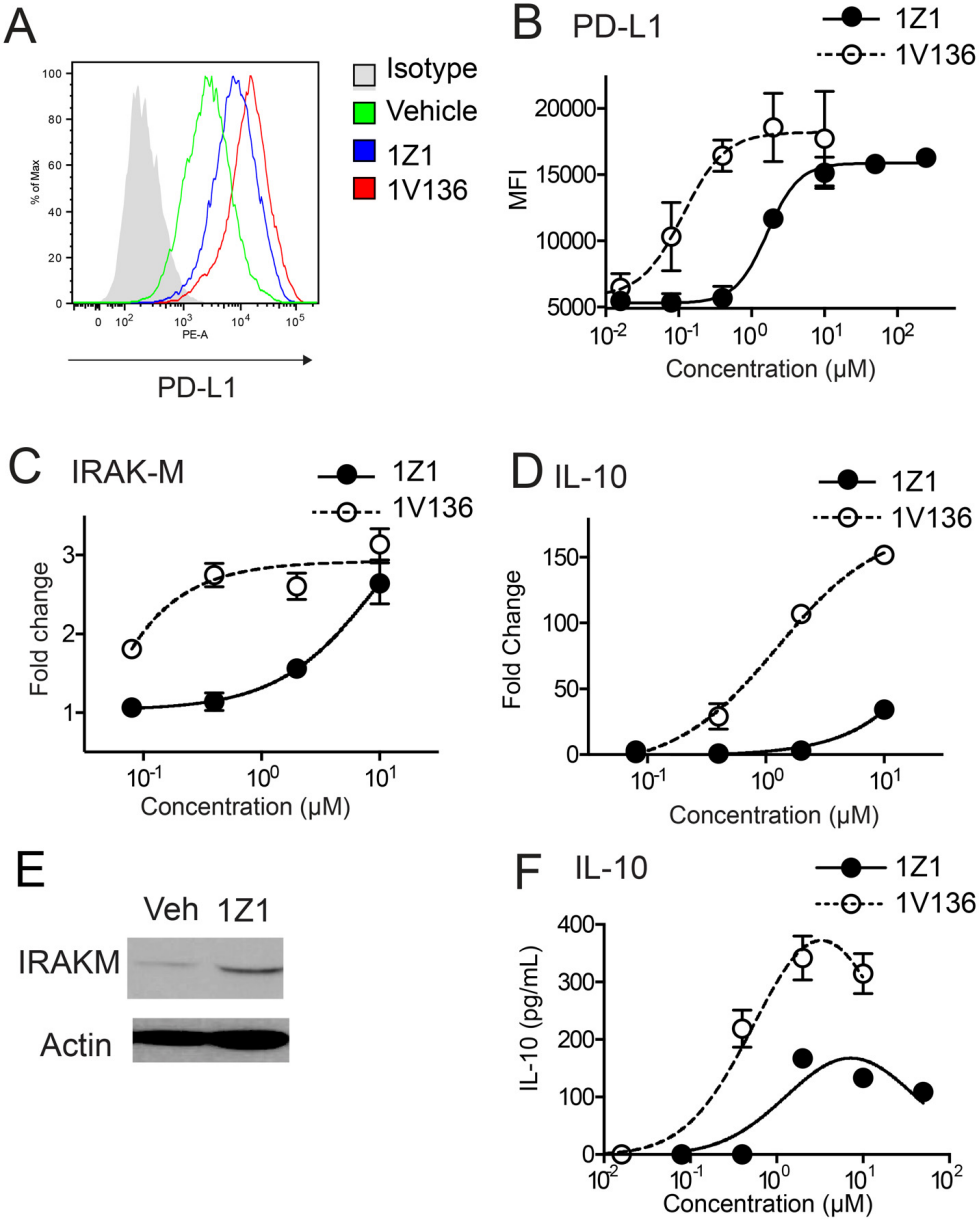
1Z1 treated DC express cell surface and intracellular regulatory inhibitors. (A and B) BMDC from C57BL/6 were incubated with graded concentrations of 1Z1 overnight and PD-L1 expression was measured by flow cytometry. Examples of PD-L1 induction by vehicle (green), 1Z1 (blue) and 1V136 (red) are shown (A) with the isotype control antibody in gray. (B) The average MFI ± SEM of triplicates are shown. These results were replicated in three independent experiments. (C and D) BMDC were treated with 1Z1 or 1V136 overnight in triplicate and mRNA levels of IRAK-M (C), and IL-10 (D) were evaluated by qRT-PCR. Fold increases of expression by graded concentration of 1Z1 and 1V136 were plotted. Data shown is the average fold induction ± SEM of triplicate and are representative of 2 independent experiments. (E) Induction of IRAK-M protein in the cell lysates from 1Z1 or 1V136 treated cells was detected by immunoblotting. (F) BMDC were treated with graded doses of 1Z1 and 1V136 as above and the levels of IL-10 in the supernatant were measured by ELISA. Data shown is the average ± SEM of triplicate and are representative of 2 independent experiments.

To examine the mechanism of the suppressive effect observed with the transfer of 1Z1 pretreated BMDC, we evaluated the induced expression of several potential mediators. 1Z1 treatment enhanced surface expression of PD-L1 on BMDC as measured by flow cytometry (Fig 4A and 4B). Furthermore, we also evaluated expression of the interleukin-1 receptor-associated kinase M (IRAK-M), which is an established negative regulator of TLR signaling [31, 32]. 1Z1 treatment induced IRAK-M in a dose dependent manner, as assessed by quantitative RT-PCR (Fig 4C), and expression was confirmed by immunoblotting after 1Z1 treatment (Fig 4E and S3 Fig). The mRNA expression for IL-10 was also examined. Expression of IL-10 increased (Fig 4D), but was less than that induced by the reference TLR7 ligand 1V136 (Fig 4F). The expression of PD-L1 and IRAK-M may be implicated in the ability of the 1Z1 treated BMDC to temporarily attenuate the onset of insulitis in the NOD mice.

### Daily administration of 1Z1 delays onset of diabetes in NOD mice

**Fig 5:**
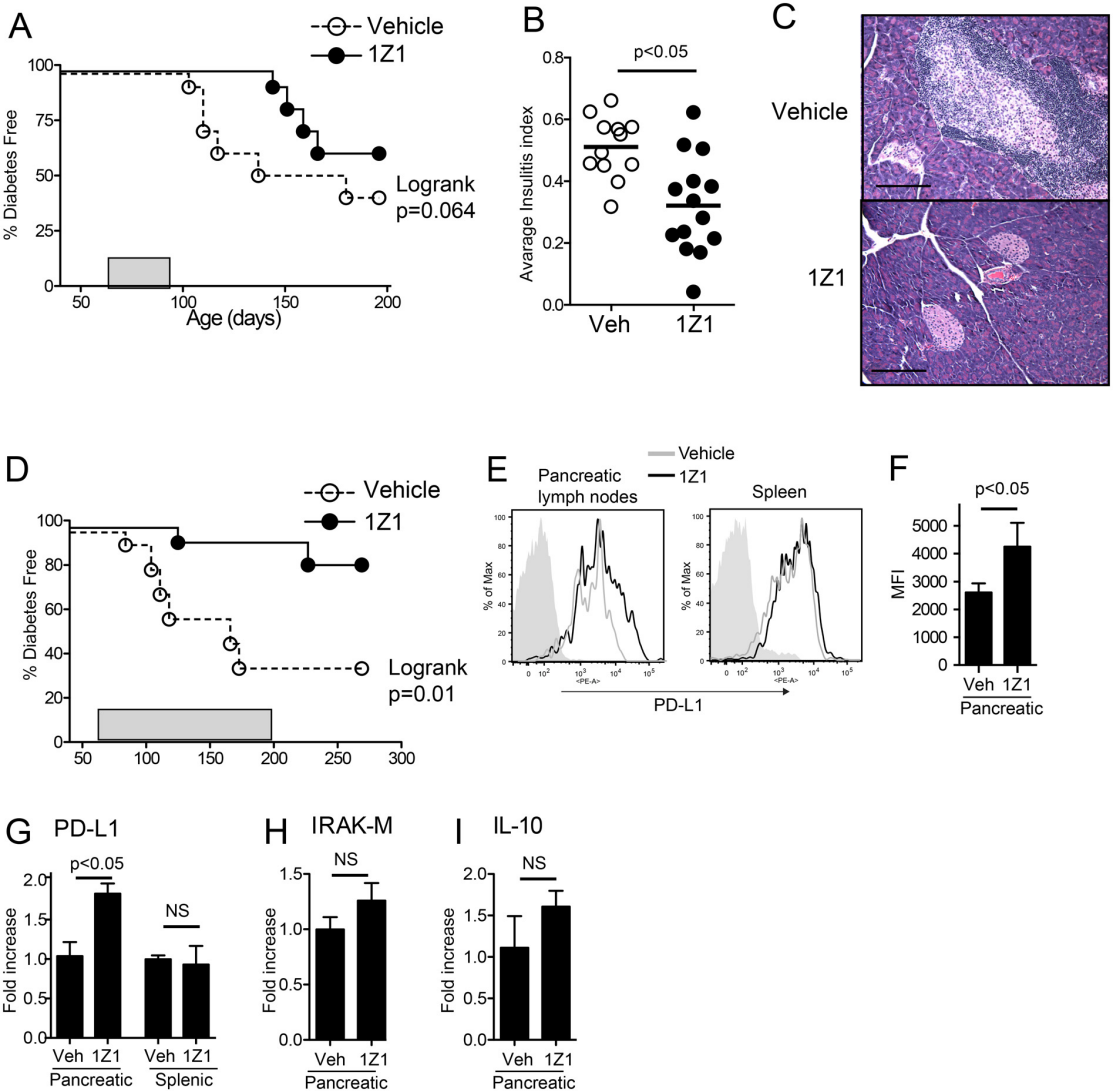
*In vivo* 1Z1 treatment reduces the progression of insulitis and activation of T cells in local lymph nodes. (A) NOD mice (n = 10) were s.c. treated daily with 1Z1 from 8 to 12 weeks of age (indicated by the gray box) and diabetes incidence was monitored to 28 weeks of age. (B) Pancreases of NOD mice treated with vehicle or 1Z1 for 4 weeks were harvested and stained with H&E, and severity of insulitis was evaluated. Ten to 40 islets from each pancreas were scored. The scoring and calculation of insulitis indexes were described in the Material and Methods. Data shown are representative of two independent experiments that had similar results. (C) Representative pancreas islets stained with H&E. Original magnification × 200. The bar indicates 200 μm. (D) Diabetes incidence of NOD mice (n = 10) s.c. treated with vehicle or 1Z1 starting at 9 weeks of age. The mice were treated for 18 weeks, indicated by the gray box, and monitored up to 38 weeks of age. (E) Representative surface expression of PD-L1 on CD11c^+^ gated cells from the peri-pancreatic draining lymph nodes and spleens pooled from NOD mice (n = 3–5 /group) treated daily with vehicle or 400nmol 1Z1. and (F) the average MFI ± SEM for 6 independent experiments is shown. The mRNA transcripts for (G) PD-L1, (H) IRAK-M and (I) IL-10 were measured by quantitative RT-PCR. Shown are the averages± SEM pooled from three independent experiments.

Dendritic cell based therapy has been reported as a treatment for T1D and other autoimmune diseases [33–35]. Clinical development of tolerogenic DC based therapy has been limited by technical difficulties and safety concerns related to generation of clinical grade tolerogenic DC. Because our data indicated that 1Z1 induced functionally tolerogenic DC without causing inflammatory responses both *in vitro* and *in vivo* (Fig 1 and Fig 3), we hypothesized that systemic 1Z1 administration could attenuate pancreatic insulitis and prevent islet destruction after adaptive autoimmunity had been established. To test this hypothesis, prediabetic 8–9 week old female NOD mice were treated with a daily dose of 400 nmoles 1Z1 s.c. [17], starting at 8 weeks of age (56 days) for 4 weeks, and monitored for diabetes onset (Fig 5). This dose was selected as highest dose that did not induce any cytokine induction in NOD mice (Fig 1D). Four weeks of treatment with 1Z1 delayed the onset of diabetes in NOD mice by 40 days (Fig 5A), but the delay was not significant (p = 0.064 by log rank test). Histologic examination of the pancreases at the end of treatment (12 weeks of age, 85 days) showed significantly reduced insulitis in the 1Z1 treated mice (p = 0.03) (Fig 5B and 5C). Furthermore, continuous treatment with 1Z1 for 18 weeks significantly protected mice from diabetic onset (Fig 5D, p = 0.01 by log rank test).

The 1Z1 treatment may be very well-tolerated without any discernible side effects. We evaluated body weight changes and serum cytokine levels in the NOD mice after the 1^st^ and the 7^th^ treatments with 1Z1 (S4 Fig). The therapeutic regimen did not change the body weights (S4 Fig) and there was no detectable increase in cytokines in the sera (S4 Fig). These data suggest that long-term administration of 1Z1 is relatively safe to the recipient.

### 1Z1 treatment induces PD-L1 expression by local dendritic cells

To investigate the mechanism of action of 1Z1 in prediabetic NOD mice, pancreatic lymph nodes were harvested from animals that had been treated with daily 1Z1 (400nmol) or vehicle for one week. Flow cytometric assays revealed an increase in the expression of PD-L1 on the gated CD11c^+^ DC cells from the pancreatic lymph nodes of 1Z1 treated mice, but not in the splenic CD11c^+^ cells (Fig 5E). The average MFI of surface PD-L1 expression in the pancreatic lymph node CD11c^+^ DC population from 1Z1-treated mice were significantly higher than vehicle treated mice (Fig 5F). Expression of other co-stimulatory molecules (CD40, CD80, CD86, or MHC class II) were not increased in the pancreatic lymph nodes of 1Z1 treated mice (S5 Fig). The upregulation of PD-L1 was concordant with the rise in PD-L1 mRNA expression in pancreatic lymph node cells and splenocytes, as assessed by quantitative RT-PCR assay (Fig 5G). However the amplicons for IRAK-M and IL-10 were not increased in the same samples (Fig 5H and 5I).

The 1Z1 treatment did not alter the relative frequencies of CD4^+^, or CD8^+^ T cells, nor increase the Treg population (CD25^+^/Foxp3^+^) in the gated CD4^+^ T cells in the pancreatic lymph nodes (S6A and S6B Fig respectively). The 1Z1 treated mice also had a reduced proportion of activated (CD69^+^) CD4^+^ T cells in the pancreatic lymph nodes (vehicle- vs. 1Z1-treated; 21% vs. 11% respectively), while similar proportions of activated T cells were detected in the spleens (5.3% vs. 6.5%, respectively) (S6 Fig).

### Minimal influence of 1Z1 treatment on regulatory T cells in vivo

Recent data suggest that TLRs are also expressed on CD4^+^ T cells and CD4^+^CD25^+^ Tregs [36, 37]. DC exposure to a TLR2 ligand enhanced CD4^+^CD25^+^ Treg proliferation, and treatment of pre-diabetic mice with a synthetic TLR2 agonist diminished the onset of T1D, and increased the number and function of CD4^+^CD25^+^ Treg [30, 38, 39]. Hence, involvement of Treg in the anti-inflammatory effect induced by 1Z1 in the NOD mice was studied to address whether 1Z1 directly stimulates Treg,

To test whether 1Z1 directly stimulated Treg proliferation, purified CD4^+^ T cells were incubated with 1Z1 in the presence or absence of anti-CD3 antibody. The stimulatory activity was measured by CFSE dilution (S7 Fig). Proliferation required CD3 engagement and was not stimulated by 1Z1 alone. In the presence of anti-CD3, the two higher doses of 1Z1 (5 and 10 μM) enhanced cell division of CD4^+^CD25^+^ T cells, but not the lower doses of 1 and 2 μM) (S7 Fig).

Next, to study whether *ex-vivo* generated BMDC treated with 1Z1 are able to induce Treg, the number of Treg in the spleens or pancreatic lymph nodes of recipient NOD mice was assessed by flow cytometry seven days after transfer of 1Z1(2 μM)-treated BMDC (S8 Fig). There was no increase in the CD4^+^/CD25^+^/CD127^lo^ Treg population between the splenocytes of the 1Z1-DC recipient NOD mice compared to controls (53.1 ± 2.5% vs. 42.1 ± 1.4%)[40, 41]. The Treg population in the pancreatic lymph nodes of 1Z1-DC recipient NOD mice and controls were similar between the two groups (69.1 vs. 65.2%).

Lastly, the suppressive function of Treg isolated from 1Z1 or vehicle treated NOD mice were compared (S9 Fig). NOD mice were treated with 400 nmol 1Z1 or vehicle every day for one week. The splenic Treg cells isolated from the mice were plated with Tconv cells at a 5:1 ratio with anti-CD3/CD28 stimulatory beads. The Treg cells from vehicle treated NOD mice reduced the percent proliferation of Tconv cells by 5.5% (28.6 vs 23.1%). Similarly the Treg from 1Z1 treated mice suppressed Tconv proliferation 5.4% (25.9 vs 20.5%) (S9 Fig). These results suggest that the induction of Treg cells was not the dominant effect of chronic 1Z1 treatment *in vivo*.

## Discussion

Type 1 diabetes (T1D) is induced by auto-reactive T cells that attack beta cells in the pancreatic islets. Although the pathogenesis of this disorder depends on adaptive T cell immune responses, DC activation plays an important role in the onset and progression of the disease [42–45]. DC are professional antigen presenting cells (APCs) that are involved in both arms of immune regulation: activation and modulation. Upon receiving danger signals and exogenous antigens, DC initiate inflammatory responses to induce adequate immune protection. Simultaneously DC suppress excessive and unnecessary immune responses to prevent harmful reactions in healthy hosts [2]. In this report, we demonstrated that 1Z1, a PEGylated TLR7 ligand, stimulated minimal inflammatory responses from DC, but rather induced a functionally suppressive phenotype that delayed the onset of insulitis in prediabetic NOD mice.

Prior reports have indicated that treatment with TLR ligands or TNFα could induce a suppressor phenotype in DC [39, 46–50]. Here, 1Z1 treated DC exhibited a semi-mature phenotype as indicated by surface activation markers including CD40, MHC class II, CD80 and CD86. Most importantly, these DC exhibited suppressor effects on antigen-specific T cell proliferation. *In vitro* studies indicated that 1Z1 was a potent inducer of negative immune regulators, such as PD-L1, IRAK-M, or IL-10, compared to the immunostimulatory cytokine, IL-6. However, there was no detectable expansion of Treg cells in vivo in 1Z1 treated mice.

Adoptive transfer of tolerogenic DC has been successful in reducing the incidence of diabetes in NOD mice [51–54]; however, the stability of tolerogenic DC function *in vivo* after transfer has not been easily achieved. Exposure of DC to 1Z1 *ex vivo* induced robust PD-L1 expression, and 1Z1 treated cells were then able to reduce antigen specific T cell proliferation. However, a single transfer of 1Z1 treated DC was not sufficient to abrogate the onset of diabetes.

An alternative strategy to DC therapy is to induce the same PD-L1 expressing DC phenotype *in vivo* by treating NOD mice with a DC modulating drug. In NOD mice, infiltration of lymphocytes and macrophages into the pancreatic islets begins at 4 to 5 weeks of age followed by overt diabetes beginning at approximately 12 weeks of age [55]. The therapeutic application of 1Z1 beginning at 8 to 9 weeks, a time point after the usual emergence of autoreactive T cells, reduced the incidence of diabetes in NOD mice. The progression from islet cell injury to hyperglycemia requires continued beta cell destruction by autoreactive T cells. There was a sustained delay in the onset of diabetes after 1Z1 treatment withdrawal similar to the honeymoon period of human T1D [56], suggesting that islet destruction by autoreactive T cells was curtailed.

Activation of innate immune pathways by TLR ligands has been reported to inhibit progression of type 1 diabetes [30, 38, 39, 43, 50]. Recent data suggest that stimulation of TLRs can directly expand CD4^+^CD25^+^ Treg that inhibit the onset or progression of autoimmune diseases [30, 36, 37, 38]. Our data show that 1Z1 augments Treg proliferation *in vitro* in the presence of anti-CD3, but not independently. However, neither repeated injection of 1Z1 nor adoptive transfer with 1Z1 treated DC increased the frequency of Treg cells in the pancreatic lymph nodes or spleens. Furthermore, Treg isolated from NOD mice after repeated administration of 1Z1 showed similar suppressive effects as the Treg isolated from vehicle treated mice. This result suggests that the mechanisms of treatment with a TLR7 ligand is different than from those previously reported for a TLR2 ligand [38].

1Z1 induced inhibitory molecules such as IL-10, IRAK-M, and PD-L1. In 1Z1 treated NOD mice, increased PD-L1 expression on DC was detected in local pancreatic lymph nodes (Fig 5E). Furthermore, the expression of PD-L1 was limited to pancreatic lymph nodes, indicating that the treatment was active at the local site of disease. This was not a global effect as splenic DC did not have any change in PD-L1 expression. The difference might be attributable to distribution, as 1Z1 is a PEGylated compound. Inflamed areas have hyper-permeable vasculature and poor lymphatic drainage, which passively increases the retention of PEGylated compounds at sites of inflammation [57].

TLR agonists have been successfully used in NOD mice to delay T1D by inducing tolerogenic responses [38, 39, 50]. A major concern with the clinical application of TLR agonists is that exposure to a sufficiently high concentration of a TLR ligand might lead to an in vivo cytokine storm. Indeed, cytokine release syndrome (CRS) is one of the most frequently occurring adverse effects of immunomodulatory agents [58]. By adding the PEGylated moiety we hypothesized that the TLR7 ligand would have a reduced rate of accumulation in the intracellular endosomal compartment, where TLR7 is functional, and therefore would limit a synchronized TLR7 signal that might be required for a sudden release of cytokines. Our previous and current studies indicated that 1Z1 induced minimal proinflammatory cytokines after *in vivo* administration, and did not induce any weight loss [16], in contrast to unmodified TLR7 agonists [59]. Furthermore, 1Z1 did not cause splenocyte proliferation up to a dose of 5 μM (Fig 1). It is possible that PEGylation of the TLR7 ligands reduced protein binding required for uptake by B cells (but not DC) [16]. Thus the PEGylated TLR7 ligand had improved safety at the cellular level by reducing access to intracellular TLR7 in the endosomes of B cells, but not in macropinocytic cells like DC.

In summary, 1Z1 induced a suppressive DC phenotype *in vivo* in the local pancreatic lymph nodes, characterized by effective induction of negative feedback molecules, including PD-L1. 1Z1 has bioactivity in the draining lymph nodes of inflamed organs, but induces minimal adverse proinflammatory cytokines and B cell proliferation. 1Z1 may be a new class of drug that selectively induces suppressive DC in local lymphoid organs with resulting benefit in treatment of autoimmune diabetes and possibly other autoimmune diseases.

## Supporting Information

S1 Fig High dose 1Z1 induces costimulatory molecules at a level similar to low dose of 1V136. (PDF)

S2 Fig Islet antigenic peptide pulsed 1Z1 treated DC accelerates insulitis. (PDF)

S3 Fig Induction of IRAK-M protein in 1Z1 treated BMDM. (PDF)

S4 Fig 1Z1 treatment does not cause weight loss or cytokine storm. (PDF)

S5 Fig CD80, CD86, CD40, and MHC class II expression in CD11c^+^ gated population in pancreatic lymph nodes after 1Z1 treatment. (PDF)

S6 Fig T cell population in spleens from vehicle- or 1Z1 treated NOD mice. (PDF)

S7 Fig 1Z1 induces proliferation of Treg only in the presence of CD3 engagement. (PDF)

S8 Fig Administration of 1Z1 treated DC does not increase Treg population. (PDF)

S9 Fig 1Z1 treatment does not enhance the suppressor function of Treg. (PDF)
