# Supplementary material for: Induction of Tolerogenic Dendritic Cells by a PEGylated TLR7 Ligand for Treatment of Type 1 Diabetes
Source: PLoS One. 2015 Jun 15;10(6):e0129867. doi: 10.1371/journal.pone.0129867 (PMC4468074; doi:10.1371/journal.pone.0129867)
Supplement: S1 Fig — (PDF) [file pone.0129867.s001.pdf]

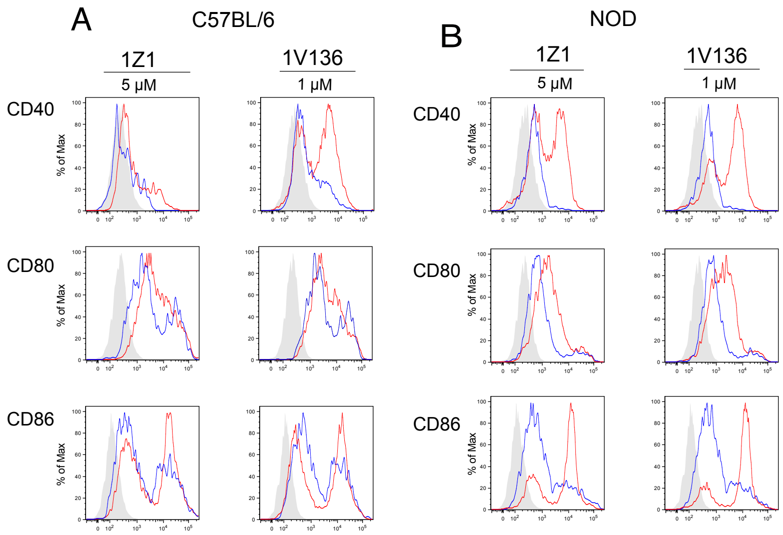

Supplemental Fig. 1. High dose 1Z1 reduces costimulatory molecules at a level similar to low dose of 1V136. WT BMDC prepared from C57BL/6 (A) and NOD mice (B) were incubated with vehicle, 1Z1 (5μM) or 1V136 (1 μM) overnight. The expression levels of CD40, CD80, and CD86 in the gated CD11c^{+} population are shown (solid red line). The expression of vehicle treated cells and isotype staining are shown as a blue line and shaded gray, respectively. Representative histograms are shown. Similar results were seen in 3 independent experiments.
