# Supplementary material for: Induction of Tolerogenic Dendritic Cells by a PEGylated TLR7 Ligand for Treatment of Type 1 Diabetes
Source: PLoS One. 2015 Jun 15;10(6):e0129867. doi: 10.1371/journal.pone.0129867 (PMC4468074; doi:10.1371/journal.pone.0129867)
Supplement: S2 Fig — (PDF) [file pone.0129867.s002.pdf]

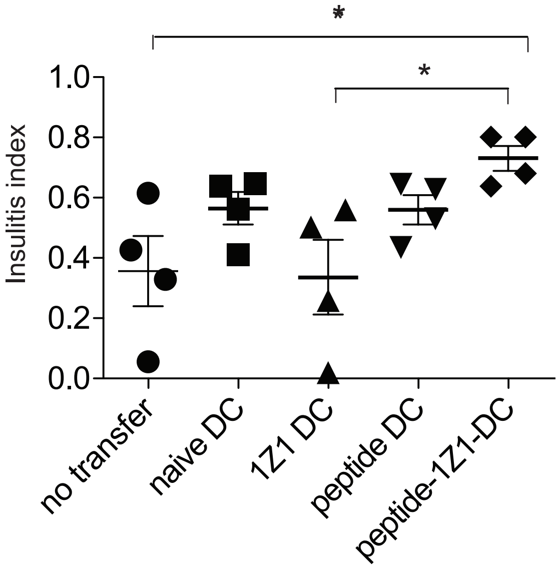

Supplemental Fig.2. Islet antigenic peptide pulsed 1Z1 treated DC accerelated inslitis. NOD BMDC were pulsed with the islet peptide $GAD65_{515\text{-}524}$ (100 μg/mL) with or without 1Z1 for 5 hours and then transferred into 8 week old female NOD mice (4/group). Vehicle treated DC and 1Z1 treated DC were used as controls. Nine weeks after DC transfer, mice were sacrificed and insulitis index in the pacreatic tissues were examined.
*:p,0.05 between indicated two groups by one way ANOVA and Bonferroni *post hoc* testing.
