# Supplementary material for: Induction of Tolerogenic Dendritic Cells by a PEGylated TLR7 Ligand for Treatment of Type 1 Diabetes
Source: PLoS One. 2015 Jun 15;10(6):e0129867. doi: 10.1371/journal.pone.0129867 (PMC4468074; doi:10.1371/journal.pone.0129867)
Supplement: S3 Fig — (PDF) [file pone.0129867.s003.pdf]

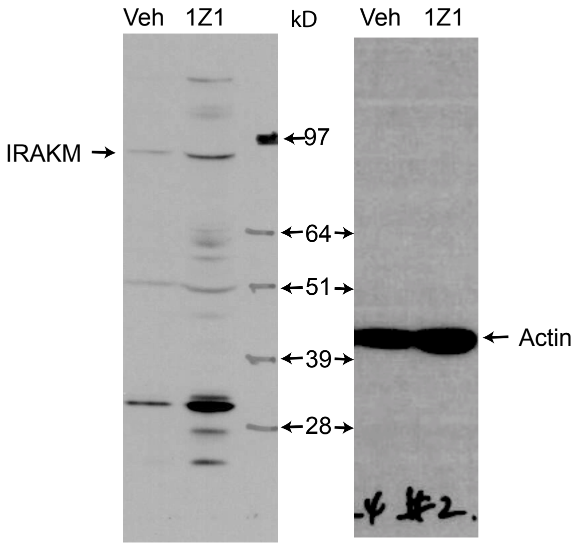

Supplemental Fig.3. Induction of IRAK-M protein in 1Z1 treated BMDM. BMDM (10^6) were stimulated with 10 μM 1Z1 or vehicle overnight. Cell lysates were separated by 4~12% SDS-PAGE gel. After the proteins were transfered onto a PVDF membrane, immunoblot analysis was performed with anti-IRAK-M or anti-actin antibodies.
