# Supplementary material for: Induction of Tolerogenic Dendritic Cells by a PEGylated TLR7 Ligand for Treatment of Type 1 Diabetes
Source: PLoS One. 2015 Jun 15;10(6):e0129867. doi: 10.1371/journal.pone.0129867 (PMC4468074; doi:10.1371/journal.pone.0129867)
Supplement: S4 Fig — (PDF) [file pone.0129867.s004.pdf]

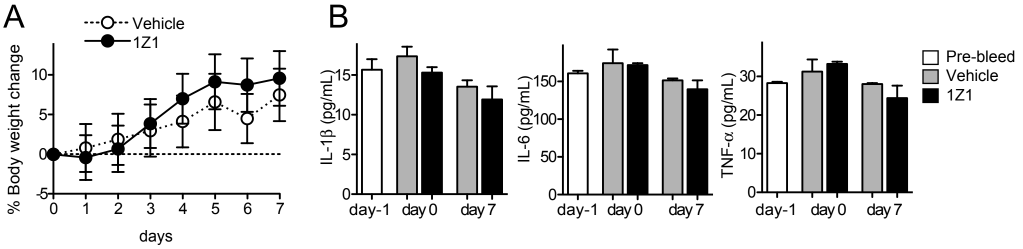

Supplemental Fig. 4 1Z1 treatment does not cause weight loss or cytokine storm. NOD mice (8 week old, n=10) were treated s.c. daily with 400 nmol 1Z1. (A) Body weight was monitored daily and expressed as a % change of the initial body weight. Average initial body weights of vehicle- and 1Z1-treated mice were 18.8 ± 0.4 g and 18.4 ± 0.4 g, respectively. (B) The sera were collected 2 h after the initial and 7th injection and levels of IL-6, and TNFa were determined by Luminex beads assay.
