# Supplementary material for: Induction of Tolerogenic Dendritic Cells by a PEGylated TLR7 Ligand for Treatment of Type 1 Diabetes
Source: PLoS One. 2015 Jun 15;10(6):e0129867. doi: 10.1371/journal.pone.0129867 (PMC4468074; doi:10.1371/journal.pone.0129867)
Supplement: S5 Fig — (PDF) [file pone.0129867.s005.pdf]

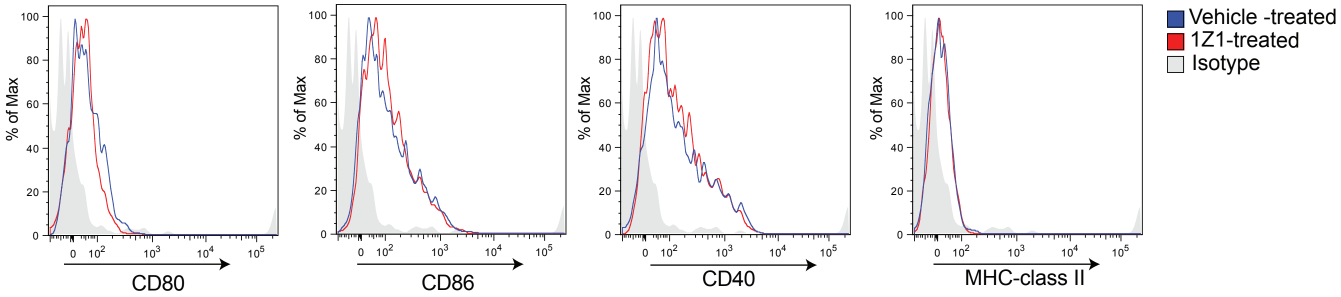

Supplemental Fig. 5. CD80, CD86, CD40, and MHC class II expression in CD11c^{+} gated population in pancreatic lymph nodes after 1Z1 treatment. NOD mice (n=3-5 /group) were daily treated with1Z1 or vehicle s.c., and the peri-pancreatic draining lymph nodes and spleens were harvested after one week after treatment. The cells were pooled in each group. Surface expression of CD80, CD86, CD40, or MHC class II was measured by FACS. Histograms shown are representatives from 3 independent experiments. Shaded gray histogram: isotype antibody staining. Blue line: vehicle treated mice. Red line:1Z1 treated mice.
