# Supplementary material for: Induction of Tolerogenic Dendritic Cells by a PEGylated TLR7 Ligand for Treatment of Type 1 Diabetes
Source: PLoS One. 2015 Jun 15;10(6):e0129867. doi: 10.1371/journal.pone.0129867 (PMC4468074; doi:10.1371/journal.pone.0129867)
Supplement: S6 Fig — (PDF) [file pone.0129867.s006.pdf]

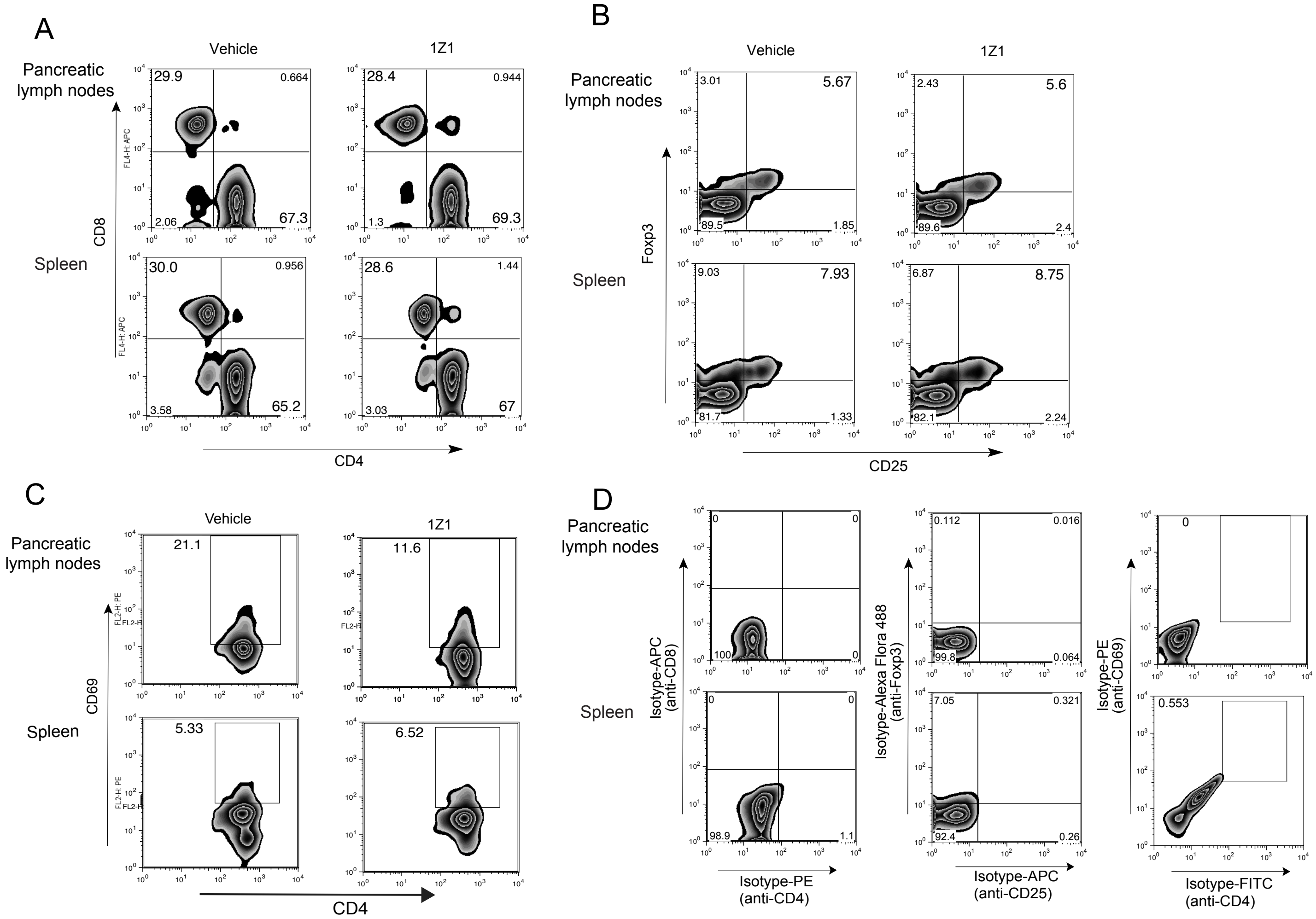

Supplemental Fig. 6. T cell population in spleens from vehicle or 1Z1 treated NOD mice.
NOD mice were daily s.c. treated with 1Z1 or vehicle for 4 weeks from 8 to 12 weeks of age (n=4-5/group). Pancreatic lymph node cells and splenocytes were collected and pooled. The cells were stained for (A) CD4 and CD8, and B) CD4, CD25 and intracellular Foxp3. $CD25^{+}$/$Foxp3^{+}$ cells were identified in the gated $CD4^{+}$ population. C) CD69 expression was evaluated in the gated $CD4^{+}$ population. (D) Isotype controls (Isotype Ig-APC, -PE, -Alexa Flora 488, or -FITC). Representative histograms are shown from 2 independent experiments.
