# Supplementary material for: Induction of Tolerogenic Dendritic Cells by a PEGylated TLR7 Ligand for Treatment of Type 1 Diabetes
Source: PLoS One. 2015 Jun 15;10(6):e0129867. doi: 10.1371/journal.pone.0129867 (PMC4468074; doi:10.1371/journal.pone.0129867)
Supplement: S7 Fig — (PDF) [file pone.0129867.s007.pdf]

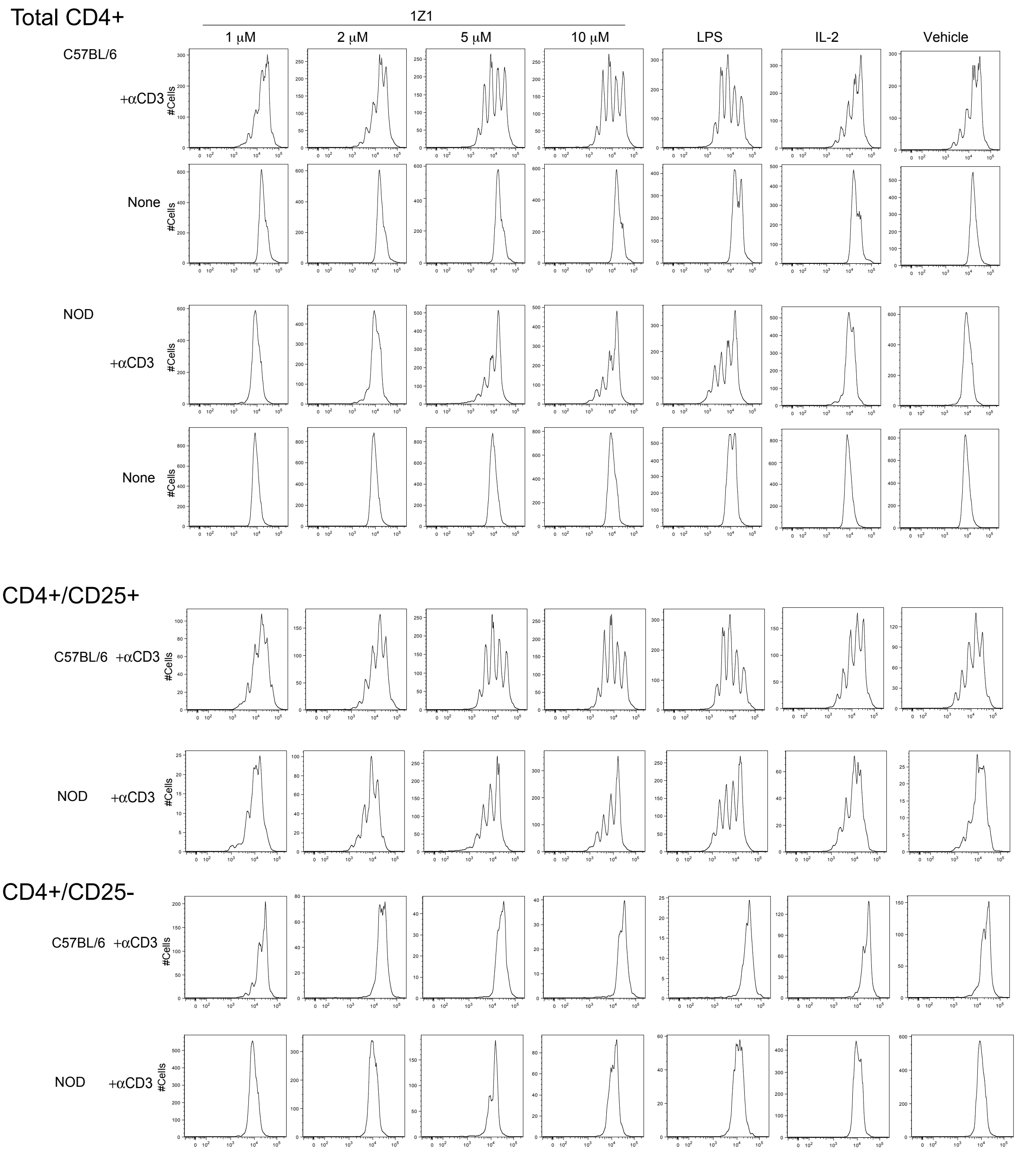

Supplemental Fig.7. 1Z1 induces proliferation of Treg only in the presence of CD3 engagement. $CD4^{+}$ T cells were isolated from splenocytes of C57BL/6 or NOD mice. CFSE-labeled CD4 T cells (10^{5}/ well in 96 well plate) were cultured with 1Z1 (1, 2, 5, or 10 μM), LPS (10 ng/mL), IL-2 (10 unit/mL) in the presence and absence of plate coated anti-CD3 monoclonal antibody (αCD3) for 3 days. The cells were stained for CD4 and CD25. Cell proliferation was monitored by CFSE dilution in $CD4^{+}$, $CD4^{+}/CD25^{+}$, or $CD4^{+}/CD25^{-}$ gated populations. The histograms presented are representative of 2 independent experiments.
