# Supplementary material for: Induction of Tolerogenic Dendritic Cells by a PEGylated TLR7 Ligand for Treatment of Type 1 Diabetes
Source: PLoS One. 2015 Jun 15;10(6):e0129867. doi: 10.1371/journal.pone.0129867 (PMC4468074; doi:10.1371/journal.pone.0129867)
Supplement: S8 Fig — (PDF) [file pone.0129867.s008.pdf]

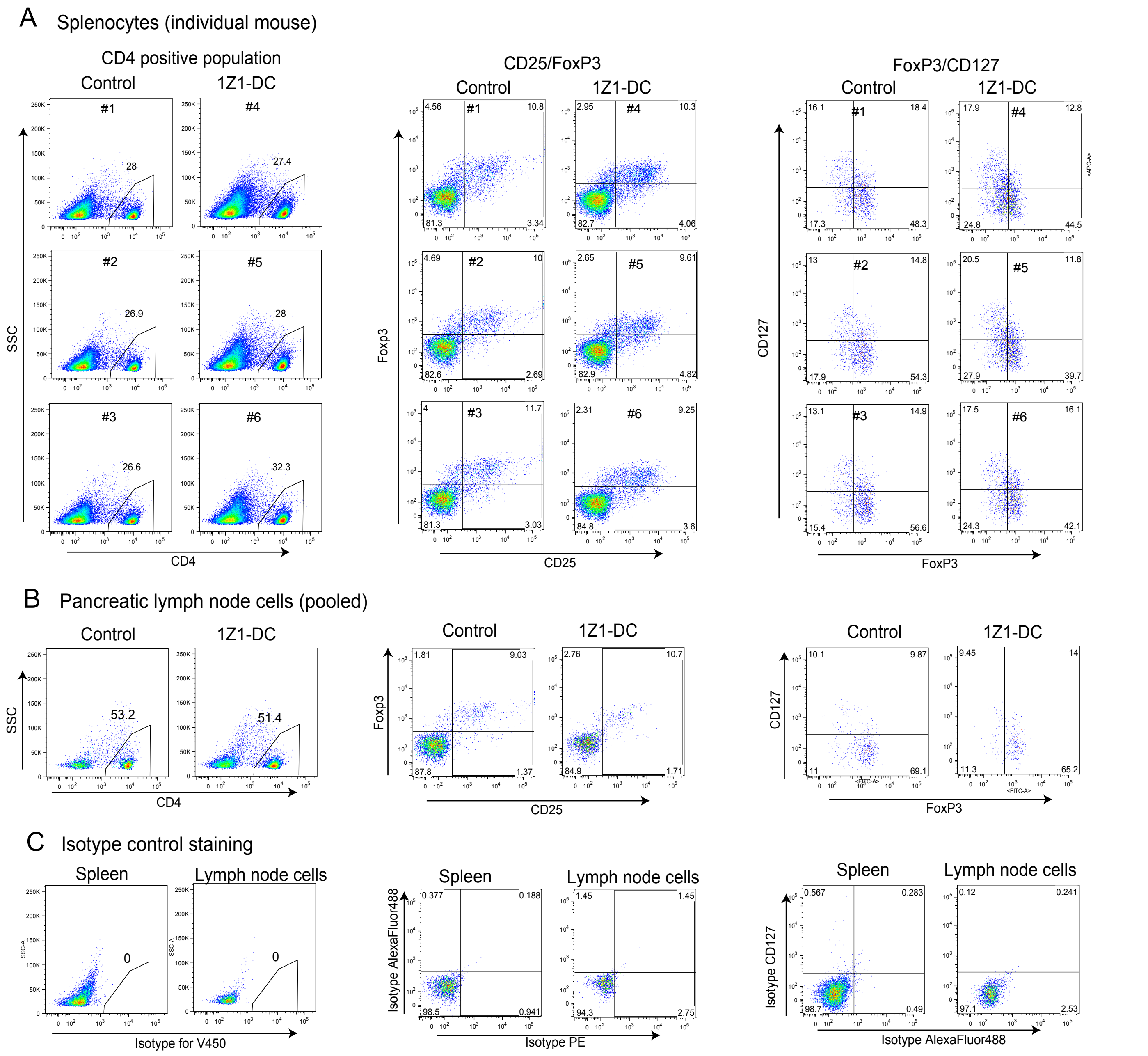

Supplemental Fig 8. Administration of 1Z1 treated DC does not increase Treg population.
BMDC were prepared from bone marrow cells isolated from 8 weeks old NOD mice. The cells were incubated with 2 µM 1Z1 overnight, washed and then adoptively transferred to 8-9 week old NOD mice (n=3/group). One week after the adoptive cell transfer, spleen and pancreatic lymph nodes were harvested. Splenocytes (A) were individually evaluated and pancreatic lymph nodes cells (B) were pooled in each group. Cells were stained for CD4, CD25, Foxp3, CD127, and isotype controls (D). The splenocyte plots are presented for each mouse from one experiment (#:mouse ID). To obtain sufficient numbers, the pancreatic lymph node cells were pooled and the plot shown is the stain of a pool of 3 mice /group. The plots shown are representative of 2 independent experiments.
