# Supplementary material for: Induction of Tolerogenic Dendritic Cells by a PEGylated TLR7 Ligand for Treatment of Type 1 Diabetes
Source: PLoS One. 2015 Jun 15;10(6):e0129867. doi: 10.1371/journal.pone.0129867 (PMC4468074; doi:10.1371/journal.pone.0129867)
Supplement: S9 Fig — (PDF) [file pone.0129867.s009.pdf]

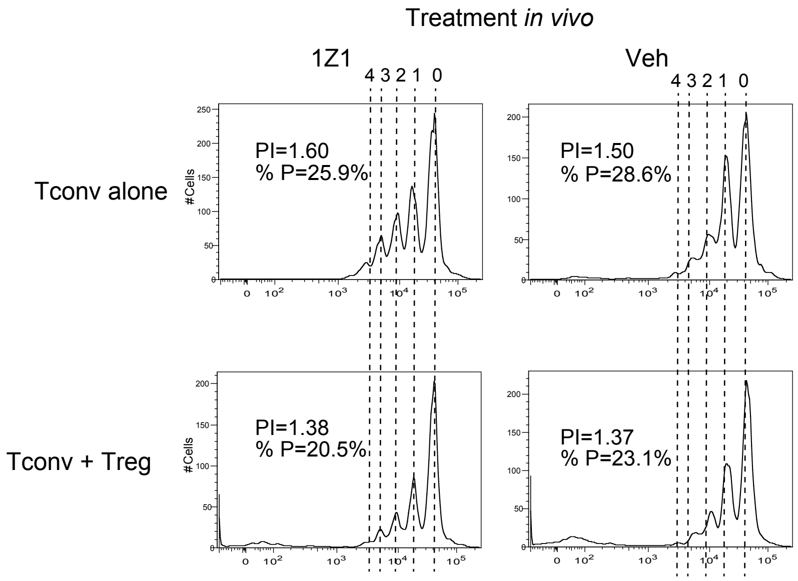

Supplemental Fig. 9. 1Z1 treatment does not enhance the suppressor function of Treg.
NOD mice were daily s.c. treated with 1Z1 (400 nmol/animal) or vehicle for 7 days. Tconv and Treg were isolated from pooled splenocytes. 1Z1 Tconv and Treg were isolated from 1Z1- or vehicle- treated mice. CFSE-labeled Tconv (10^5) were cultured alone or with Treg ($2x10^4$) in the presence of anti-CD3 and anti-CD28 antibody coated beads. Cell proliferation was monitored by CFSE dilution in CD4 gated population. Cell division was quantified by proliferative index and % proliferation as described in Materials and Methods. Data shown are representative histograms from 3 independent experiments.
